# Successful Treatment of Frozen Hip with Manipulation and Pressure Dilatation

**DOI:** 10.2174/1874312900802010031

**Published:** 2008-04-11

**Authors:** R Luukkainen, E Sipola, P Varjo

**Affiliations:** 1 Satakunta Central Hospital, Steniuskenkatu 2, 26100 Rauma, Finland; 2 Turku University Hospital, Alvar Aallontie 275, 21540 Paimio, Finland; 3 Satakunta Central Hospital, Sairaalantie 3, 28500, Pori, Finland

## Abstract

A 44-year old man with prolonged frozen hip was treated with manipulation under anesthesia and pressure dilatation of the left hip joint. The treatment was successful and after one year the hip was symptomless.

## INTRODUCTION

Frozen shoulder or adhesive capsulitis is a well-known and rather common clinical disorder [1,2]. An analogous condition in the hip joint seems, in contrast, to be very uncommon. According to earlier reports it is resistant to conventional treatment but spontaneous resolution can be expected in periods varying from 3 to 18 months [3-6]. Chard and Jenner have named this condition as frozen hip and according to them it is probably underdiagnosed [5]. We describe a case of frozen hip that was treated with manipulation and pressure dilatation.

## CASE REPORT

**Fig. (1):**
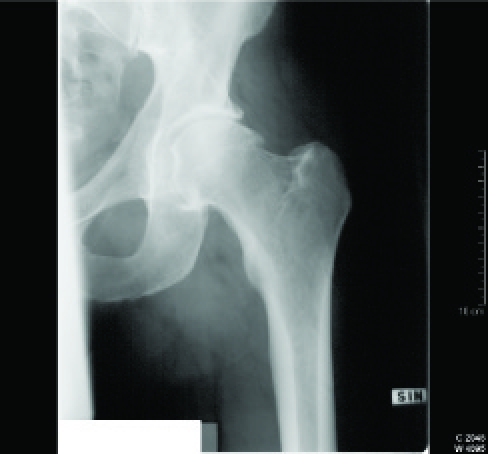
Plain radiography of the left hip.

**Fig. (2):**
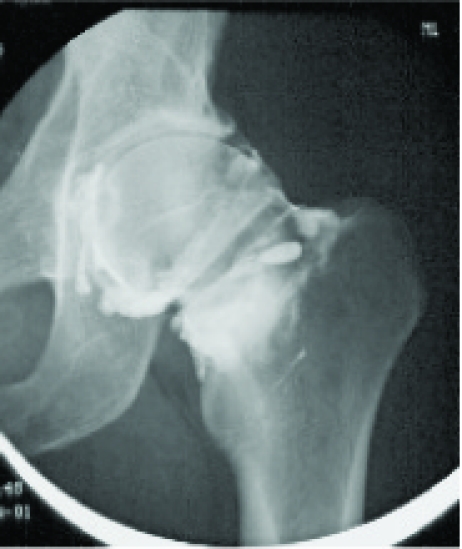
Arthrography of the left hip.

A 44-year-old man was investigated in March 2001, because his left hip had become stiff and painful without any previous infection or injury in November 2000. Before this he had been healthy and during free time he had exercised actively. On examination his overall condition was good. The flexion and rotation of his left hip were considerably restricted, while other joints were normal. Plain radiography of left hip showed only mild arthrosis (Fig.**1**) but ultrasonography and hip aspiration were normal. Articular capacity in hip arthrography (Fig.**2**) was diminished and bone scan showed mild increase in uptake. Erythrocyte sedimentation rate, C-reactive protein and haemoglobin were normal and rheumatoid factor, antinuclear antibodies and antibodies to Borrelia were negative. Frozen hip was diagnosed. The patient received physical therapy, non-steroidal anti-inflammatory drugs and the hip was injected with 1.5 ml (20 mg/ml) of intra-articular triamcinolone hexacetonide and 1.5 ml (20 mg/ml) of lidocaine. These treatments were without either subjective or objective response. Magnetic imaging resonance of the left hip was performed in October 2003 and the result was normal. The symptoms continued and disabled the patient considerably. Manipulation under anaesthesia and pressure dilatation of the joint space were performed in November 2003. The method of dilatation was by infusing isotonic sodium chloride with pressure up to 281 ml and controlling the procedure by fluoroscopy and contrast medium. Physical therapy was given afterwards and the hip became painless without any limitation in ranges of motions. The ranges of movements of the left hip before the manipulation and pressure dilatation were as following: flexion 85^o^, extension 10^o^, abduction 25^o^, adduction 15^o^, internal rotation 5^o^ and external rotation 15^o^; just after pressure dilatation and manipulation: flexion 120^o^, extension 10^o,^ abduction 35^o^, adduction 30^o^, internal rotation 25^o^ and external rotation 35^o^; and in February 2004: flexion 110^o^, extension 10^o^, abduction 40^o^, adduction 20^o^, internal rotation 20^o^ and external rotation 25^o^. In December 2004 the left hip as well as other joints were symptomless.

## DISCUSSION

According to earlier reports frozen hip is a clinical entity, which causes pain and shows limitation of active and passive ranges of movements. Investigations to exclude systemic disease must yield negative results, but an isotope scan may show an increased uptake. The treatment of frozen hip by physical therapy, non-steroidal anti-inflammatory drugs, intra-articular corticosteroids and local anesthetics has proved to be ineffective [3-6]. The condition has good prognosis and a spontaneous resolution can be expected [3-6]. Our patient fits well with the condition described, except that his disability did not disappear even within 3 years.

So, in our opinion frozen hip can be treated with manipulation and pressure dilatation under anaesthesia especially in cases when spontaneous recovery is delayed. Since for example 18 months with stiff and painful hip is a long time, this kind of therapy could be indicated even in earlier phases of the condition. However, prospective studies are necessary, before any recommendations of this kind of treatment can be given.
